# Octree Optimized Micrometric Fibrous Microstructure Generation for Domain Reconstruction and Flow Simulation

**DOI:** 10.3390/e23091156

**Published:** 2021-09-02

**Authors:** Nesrine Aissa, Louis Douteau, Emmanuelle Abisset-Chavanne, Hugues Digonnet, Patrice Laure, Luisa Silva

**Affiliations:** 1 High Performance Computing Insitute, Ecole Centrale de Nantes, 1 rue de la Noe, 44300 Nantes, France; louis.douteau@ec-nantes.fr (L.D.); hugues.digonnet@ec-nantes.fr (H.D.); 2 GeM Institute, Ecole Centrale de Nantes, 1 rue de la Noe, 44300 Nantes, France; 3 I2M Institute, CNRS, Arts et Métiers Paris Tech, University Bordeaux, Bordeaux INP, 33400 Talence, France; emmanuelle.abisset-chavanne@ensam.eu; 4 Laboratoire J.-A. Dieudonné, CNRS UMR 6621, Université de Nice-Sophia Antipolis, 06000 Nice, France; patrice.laure@mines-paristech.fr; 5 Cemef, UMR CNRS 7635, Mines ParisTech, 06904 Sophia Antipolis, France

**Keywords:** octree optimization, microstructure generation, domain reconstruction, flow simulation, permeability computing

## Abstract

Over recent decades, tremendous advances in the field of scalable numerical tools and mesh immersion techniques have been achieved to improve numerical efficiency while preserving a good quality of the obtained results. In this context, an octree-optimized microstructure generation and domain reconstruction with adaptative meshing is presented and illustrated through a flow simulation example applied to permeability computation of micrometric fibrous materials. Thanks to the octree implementation, the numerous distance calculations in these processes are decreased, thus the computational complexity is reduced. Using the parallel environment of the ICI-tech library as a mesher and a solver, a large scale case study is performed. The study is applied to the computation of the full permeability tensor of a three-dimensional microstructure containing 10,000 fibers. The considered flow is a Stokes flow and it is solved with a stabilized finite element formulation and a monolithic approach.

## 1. Introduction

The properties and behavior of a discontinuous fiber-reinforced thermoplastic are induced by the mechanisms involved during the forming process. Modeling and numerical simulation have a major role in understanding and predicting these mechanisms, especially at the microscopic scale, which provides the most accurate results. Nevertheless, at this scale of computation, numerical simulations are generally expensive in terms of computing resources and time. Optimizing and evaluating the used algorithms is a constant challenge. One of the most expensive issues when using finite elements and immersed boundary approaches for discontinuous reinforced composites simulation is the computation of distances. Fibers generation, immersion, and reconstruction techniques particularly rely on these evaluations, as the distances between fibers must be regularly evaluated during microstructure generation and distances from each point of the computational mesh to the frontiers of the immersed elements have to be measured. However, without any optimization, whenever the number of points and fibers in a simulation rises, the cost of reconstruction increases dramatically. In order to make these techniques applicable in the context of composites materials, an optimization of the distance evaluation is required. A first idea is to implement distance computation algorithms that save computational time. Reducing the number of expensive functions or operations used to compute each distance is a key element, as well as defining properly the data types used to limit memory footprint. This paper proposes a reduction in the number of distances to evaluate, which is performed using an octree.

The octree data structure [1] is a partition of a three-dimensional space built from recursive subdivisions into eight sub-domains. The sub-cubes obtained are hierarchically organized, which allows to easily reduce search time. Octree algorithms are widely used in various fields and their application range is significantly extensive, especially when positions must be accessed and manipulated. These applications include construction of a three-dimensional object model from a set of images [2] and simulation of displacement of free surface [3]. Octrees are broadly applied for collision detection algorithms in virtual reality, rigid bodies contacts, characters animation, or machining simulation, such as cutter-path generation for numerical control machines which require efficient collision detection routines [4,5,6]. Another significant example involving octree algorithm is the mesh generation procedure. Octree can be used to create meshes tied to geometrical objects [7], for adaptive mesh refinement (AMR), e.g., with structured grids in fluid dynamics [8], or combined with others techniques in advanced mesh generation processes [9].

In this paper, an octree-optimized microstructure generation and domain reconstruction with adaptative mesh is presented. An application of flow simulation through the reconstructed domains dealing with the identification of the full-component permeability tensor is conducted.

## 2. Microstructure Generation and Optimization Using Octree

The microstructure of a discontinuous fiber composite greatly affects its properties. For that, virtual numerical sample generation is crucial in order to carry out precise prediction simulations. However, a major difficulty to generate such a microstructure lies in the establishment of an optimized methodology that allows generating a very large number of fibers without interpenetration and with a minimum computation time and resources. In this work, a Random Sequential Adsorption RSA algorithm [10,11], widely used for rigid particles generation, is chosen.

A collection of *N* random unit orientations **P**, *N* homogeneously distributed mass center positions **X** and *N* lengths **L**, following a normal distribution law with mean length <L> and standard deviation σ, are primarily created. The program begins with one initial fiber (i) randomly oriented with $P_{i}$. Subsequently, another fiber (j) with a random orientation $P_{j}$ is selected and then the system is checked for overlap. If the fiber (j) intersects a pre-existing fiber, it is repositioned by randomly changing orientation vector $P_{j}$ while retaining the same position vector $X_{j}$. The selection of a new $P_{j}$ is repeated up to a maximum number of trials until the overlap condition is released. In this method, the generated geometry is periodic, so that any fiber cutting a boundary will be extended on the opposite one. This means that fibers close to surfaces can interact with the fibers of the near domains. Therefore, every new fiber to be placed is verified for interaction with all already pre-existing fibers and their 26 periodic images in the near domains.

**Figure 1:**
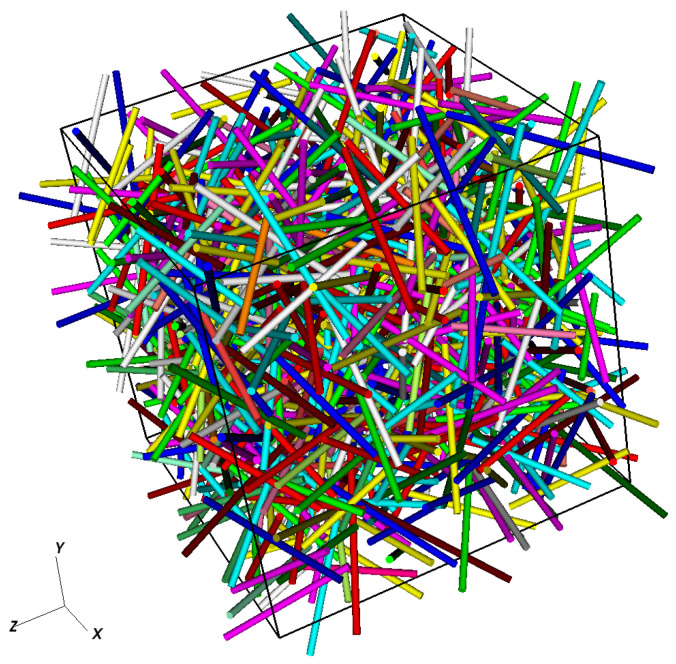
Example of a generated microstructure with 1000 fibers having a same diameter *d*, a mean aspect ratio r=<L>/d=20 and a fiber volume fraction $V_{f}=0.1$.

Figure 1 presents an example of a generated microstructure with 1000 cylindrical fibers having a same diameter *d*, a mean aspect ratio r=<L>/d=20 and a fiber volume fraction $V_{f}=0.1$.

In the previously described algorithm, N∗27 distances evaluations are required to generate the N+1-th fiber. Presuming that no intersection is detected, a minimum of 27∗N∗(N−1)/2 distances has to be computed, thus leading to a $N^{2}$ complexity. However, this number can increase, as once an intersection occurs, new random positions and orientations must be generated for the fiber. This computational cost is acceptable when *N* remains small, but becomes unaffordable when *N* reaches the order of the millions of fibers. To limit the number of distances to evaluate, this paper proposes the use of an octree algorithm. This tree structure enables to browse rapidly across all the elements and to select them based on their position. Consequently, a selection of the closest elements can be performed, which allows measuring the distances to these elements only. The complexity is decreased and can reach Nlog(N) for an optimal problem. The next paragraphs describe the octree building procedure, while the use of the octree is explained in Section 3.1.

**Figure 2:**
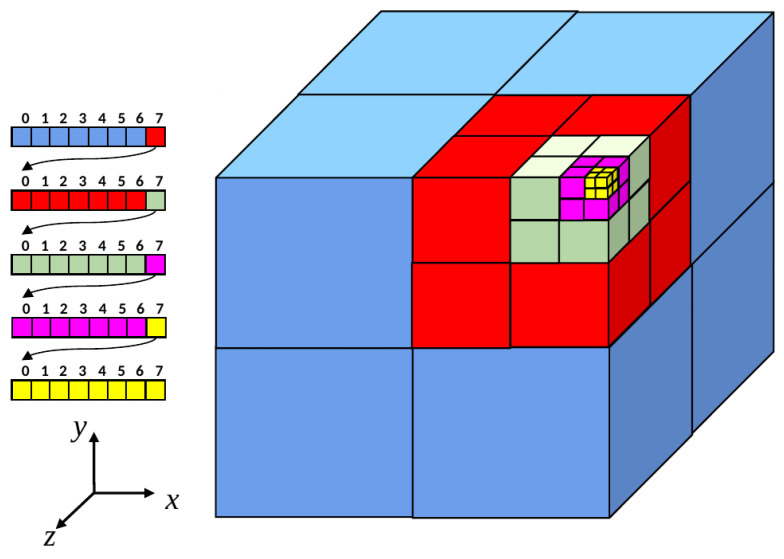
Illustration of the octree data structure: On the left is highlighted the refinement of a tree element into 8 new elements. The cube on the right presents the geometrical positions of the octree elements.

This data storage concept is a tree structure built recursively from a computational domain, in which elements, e.g., fibers, are dispersed. To clarify this paragraph, an analogy is performed between the computational domain and box bounding all the elements. In practice, there is a possibility for elements to be concentrated in a particular area of the computational domain. In that situation, the octree building procedure is processed in the interest region only, which does not cause any problem later on. The tree is built through refinement steps where the computational domain is divided in two along each dimension, thus generating subdomains (children). The name octree comes from the characterization of the tree in 3D, where 8 subdomains are generated by the division procedure (Figure 2).

After refinement, the elements shall no more be contained in the initial computational domain, but are defined using pointers towards every child they intersect. This choice characterizes the octree class, which is composed of the dimensions of the computational domain and pointers to, either the elements contained inside it or the children generated. The corollary of this choice is that fibers can be duplicated if they intersect several children. After a refinement step, all the children are overlooked with emphasis on the number of elements it contains. If a subdomain remains empty, i.e., no elements intersect it, it is immediately deleted. If too many elements are found in this child, the refinement procedure is repeated in this particular subdomain. The recursiveness is applied in that way until: either an acceptable number of elements is obtained in the deepest subdomains (leaf), or the maximal depth of the octree is reached.

The repartition of elements into the children is handled using bounding boxes. Axis-Aligned Bounding Boxes AABB have been used, which offer different advantages. First of all, these boxes are very easy to determine, both computationally speaking and in terms of access to data. It also allows reducing the computational effort for the determination of the intersections, as the boxes are oriented along the same axes as the computational domain. Finally, this choice enabled to generalize the octree to very different usage, from fibers to, e.g., 3D facets used to define surface meshes. The drawback brought by these bounding boxes lies in the intersections, as an “ill-oriented” fiber may be duplicated in leaves it does not intersect, only because its bounding box does. In that case, we can implement Oriented Bounding Box OBB in future works to enclose fibers as tightly as possible. Another limitation occurs when very long elements (proportionally to the size of the computational domain) are present, as again the fibers may be highly duplicated. However, the following developments of this paper will show that octree usage remains appropriate for elements with a small length to width ratio.

**Figure 3:**
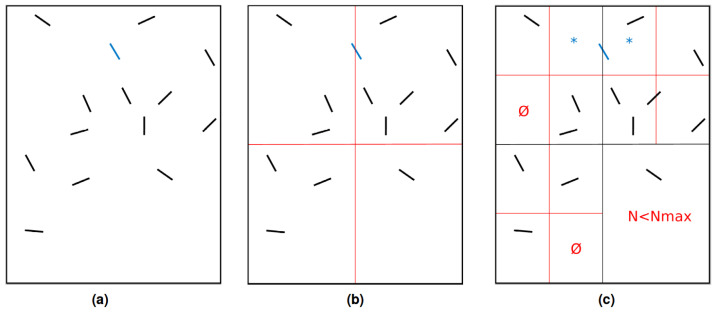
Octree generation example: (**a**) Fibers in computational domain. (**b**) Octree first level of refinement. (**c**) Octree second level of refinement.

This paragraph presents the octree generation on an example that features 14 fibers, with a maximal depth for the octree of 2 and 1 fiber allowed per leaf. The procedure is drawn in Figure 3.

The octree parameters mean that any subdomain containing more than 1 element needs to be refined, with a limit of only 2 levels. After the first step of the refinement, the fibers presented in Figure 3a are allocated to every subdomain their bounding box intersect. An interesting emphasis can be placed on the blue fiber (second “row” from the top, middle of the computational domain), which is duplicated into both of the two children on top of the initial computational domain in Figure 3. Consequently, after a second step of refinement this fiber can be found in two different octree leaves, the asterisked ones in Figure 3b. Figure 3c corresponds to the final octree as obtained with the parameters detailed previously. Even if the presence of only one fiber per leaf was authorized, subdomains containing more than one fiber can be found because of maximum refinement allowed. Note that the subdomains containing ∅ have been created by octree refinement, and immediately deleted as no fiber was allocated to it.

**Figure 4:**
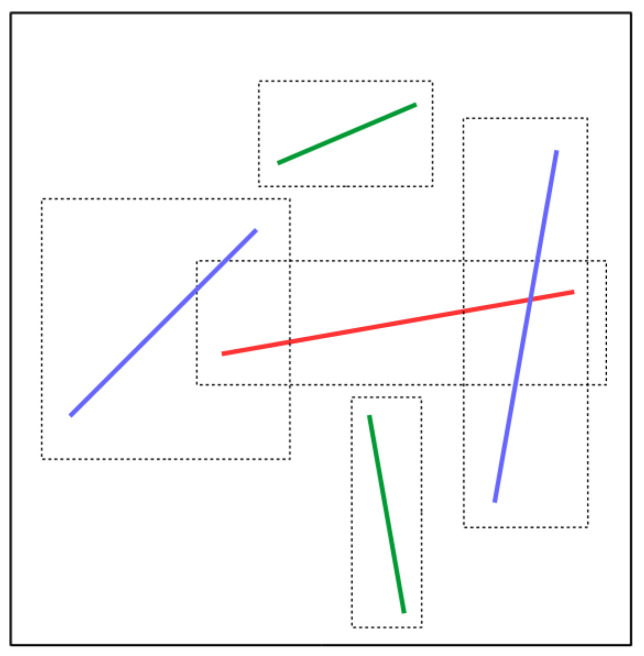
Illustration of the collision detection optimisation process: addition of the red fiber to an octree leaf, possibility of collision only with blues fibers whose **AABB**s intersect the **AABB** of the red one.

When adding a new fiber following the RSA algorithm, thanks to the implementation of the octree, the check for overlap will be carried out among a reduced number of fibers initially judged by the octree as potential candidates for collision. Fibers with which there is a possible collision are the fibers in the leaf or leaves to which the new fiber belongs and whose **AABB**s intersect. Figure 4 shows a schematic diagram of this method: it shows a leaf of an octree (large box black) to which we would like to add the red fiber and where the blue and green fibers already exist. Thus, a possibility of intersections can only occur with blue fibers. The green fibers will not be concerned because their **AABB**s do not intersect the **AABB** of the red fiber.

During this process, fibers are dynamically added to the octree. For that, two major conditions should be verified to update the octree after adding a new fiber:

- A new fiber must be always included in the global domain initially built for octree and, if it is not the case, it is necessary to destroy the octree and to reconstruct it;
- The size of a leaf should not exceed the defined maximal size and, if it is not the case, it is necessary to refine the octree.

**Figure 5:**
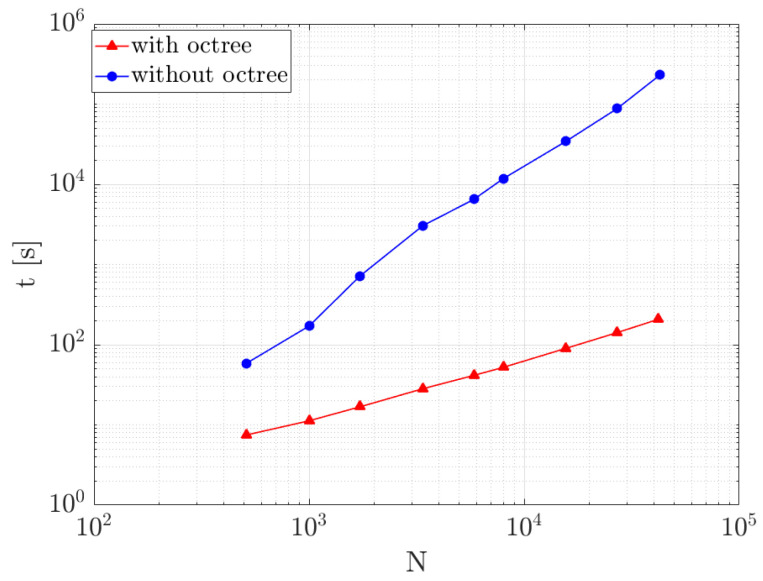
Microstructure generation time as a function of the number of fibers, for a case with microstructure having r=20, $V_{f}=0.1$, and a maximum number of trials equal to 5000.

To quantify the gain brought by the octree, we study the evolution of the CPU time, *t*, according to the number of generated fibers, *N*. For all the simulations, we consider r=20, $V_{f}=0.1$, and a maximum number of trials equal to 5000. The leaf maximal size is fixed to 100. Figure 5 shows a considerable gain on time which improves as the number of fibers becomes more important.

## 3. Computational Domain Reconstruction

### 3.1. Mesh Immersion and Optimization Using the Octree

Mesh immersion is a technique enabling the representation of complex bodies using a single computational mesh. The main idea is to compute the distance from each point of the computational mesh to an object immersed, which can be represented by an analytical function, a mesh, or any set of data.The only constraint is the need to build an interior for the object, thus defining a frontier. This definition enables to establish a signed distance function α, as presented in Equation (1) for the immersion of a shape ω of the frontier Γ=∂ω into a domain Ω. This interior can be concave or even split, as the mathematical evaluation of α does not have any prerequisite. However, the more complex ω will be, the more points in the computational mesh will be needed to represent it accurately.

(1) $$\alpha =\bar{d}\left(x,\omega \right)=\left\{\begin{array}{ccc} d(x,\Gamma ) & \mathrm{if} & x\in \omega \\ -d(x,\Gamma ) & \mathrm{if} & x\notin \omega \end{array}\right.,x\in \Omega .$$

Once the signed distance function is defined, any computational point *x* has a signed-distance either positive or negative. The union of points with positive α defines the interior, and the inverse set gives the exterior. This formulation mathematically corresponds to using a Heaviside function as a level-set function, which gives 1 for α positive and 0 for α negative. However, this approach is not suitable for multiphase flows, as strong discontinuities are sources of instability when using Galerkin approximation for the resolution of the Navier–Stokes equations. To overcome this issue, a smoothed Heaviside function based on a width parameter ε has been defined and is presented in Equation (2).

(2) $$H_{\varepsilon }\left(\alpha \right)=\frac{1}{2}\left(1+\frac{u_{\varepsilon }\left(\alpha \right)}{\varepsilon }\right),$$

with

(3) $$u_{\varepsilon }\left(\alpha \right)=\varepsilon \tanh\left(\frac{\alpha }{\varepsilon }\right).$$

This paradigm introduces a transition phase of a width of about 2ε which smooths the shifting between physical parameters of the two phases. The “blurred area” does not operate as a gray zone in terms of mesh immersion, as the norm and sign of the result given by $H_{\varepsilon }$ in this region is depending on α. Compared to immersion results giving either 0 or 1 for a classical Heaviside function, a better capture of the interfaces can even be achieved. However, the quality of the reconstruction of ω remains highly dependent on the meshing of Ω. Fine meshes are needed around interfaces, and if the meshing of ω is complex, a high effort will be put in either mesh generation or distance evaluation.

**Figure 6:**
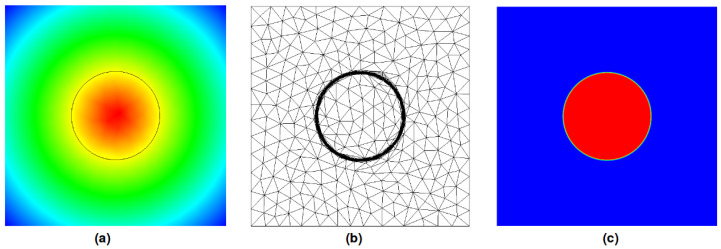
Immersion and mesh adaptation: (**a**) Signed distance α and isoline α=0. (**b**) Adapted computational mesh (**c**) Smoothed Heaviside $H_{\varepsilon }$ with ε=R/100.

This interdependency is addressed by coupling the immersion with a mesh adaptation procedure. An anisotropic mesh generated automatically concentrates its points around Γ, guaranteeing that an important portion of them will be located in the transition region highly impacted by $H_{\varepsilon }$. Further explanations about this procedure can be found in Section 3.2 and in [12]. Figure 6a presents the results of α for a circle of a radius *R*, and Figure 6b presents the results obtained for $H_{\varepsilon }$ with ε=R/100. A slice of the computational mesh is also drawn, where the major part of the points are gathered in the interest zones (Figure 6c).

The level-set function is defined analytically from α, making the evaluation of α the major effort of the immersion procedure. If an analytical definition of α requires only one distance computation per point and does not need to be optimized, considering more complex representations generates computing complexity, e.g., when meshes or fibers set are immersed. Those cases use a set of elements to define ω or Γ, so the determination of the closest neighbor is not immediate. The performance of the immersion code then highly depends on the computational effort needed to evaluate a single distance, but also on the number of distances to compute before finding the closest element of ω. Without any optimization of the immersion procedure, the computation of α for a single point *x* and *M* fibers defining ω require *M* distance evaluations. Consequently, the immersion of *M* fibers in Ω composed of *N* points forces the computation of N×M distances. When few fibers are immersed in small meshes, this cost is affordable. However, when 10,000 fibers are immersed, as proposed in the case of study of this paper, the number of computations is extremely high (assuming that *N* is quasi-linearly related to *M*), which is somewhere between not competitive and unrealizable computationally. The coupling of the mesh immersion procedure with an octree is a way to reduce the complexity. The construction of the octree was overlooked in Section 2, and its contribution to the reduction in computational costs is detailed in the next paragraphs.

Instead of computing the distance from a point *x* to each element defining ω, the idea behind the octree is to select elements located near *x*, and to compute the distance from them only. The distance computation algorithm is discussed in the following, with use of the nomenclature defined in Table 1.

All starts with the determination of the octree leaf $OL_{x}$ which is the closest from *x*. From the definition of the octree, $OL_{x}$ is proven not to be empty. Even if the closest element from *x*, named $E_{c}$, is not imperatively stored inside $OL_{x}$, its distance to *x* is inferior or equal to the distance from *x* to the closest element located inside $OL_{x}$. A well-parametrized octree guarantees that the size of the set of elements contained inside a leaf is reasonable. The distances from *x* to the bounding boxes of every element contained inside $OL_{x}$ are then computed. The distance to the furthest point of every bounding box is computed, and the minimum obtained is selected. This minimal distance $d_{x}$ defines a circle/sphere $C_{x}$ of center *x* and of radius $d_{x}$, in which the closest elements is compulsorily located. The octree is then browsed to determine all the leaves it intersects, which are candidates to host $E_{x}$. The bounding boxes of all the elements located in the selected leaves are browsed, and if the minimum distance from *x* to it is inferior to $d_{x}$, the distance from *x* to the element is computed. $\alpha_{x}$ is then obtained by selecting the minimum among the distances to elements evaluated.

Octree has been defined to be computationally efficient and stand-alone, and the use of bounding boxes is a key factor to that extent. Large computational savings are enabled as the octree only knows the elements as bounding boxes and, until the very end of the algorithm, distances computed are between *x* and the boxes. The number of distances from *x* to the elements, which can be very expensive computationally, is limited to the elements whose bounding box intersect $C_{x}$. Browsing all the boxes contained inside $OL_{x}$ to determine $d_{x}$ might seem unnecessary, but if this procedure is not completed, the maximal theoretical distance to $E_{x}$ is the distance to the furthest point of $OL_{x}$. Overlooking the boxes enables to reduce the span of $C_{x}$, which may translate to a smaller selection of octree leaves and to a reduced number of distances from *x* to elements. The computational cost of this stage, implying few distance computations to bounding boxes, often tends to be worth the savings brought by the optimization of $C_{x}$. The usage of bounding boxes also bring easy generalization of the octree procedure. The selection of the closest elements, to which distance from *x* is evaluated, is totally independent on the type of elements used. Heterogeneous sets can even be used, with, e.g., facets and fibers mixed.

**Figure 7:**
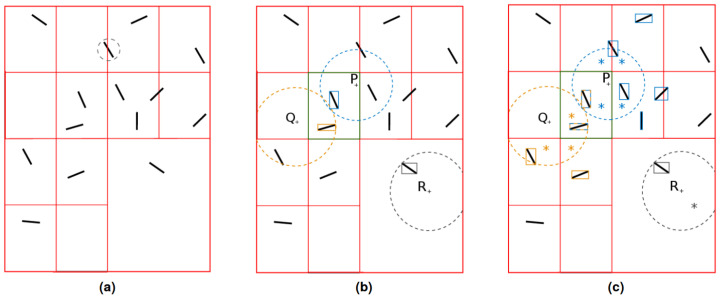
Octree fiber immersion optimization example: (**a**) Final octree. (**b**) Determination of closest leaf. (**c**) Octree leafs to consider.

Figure 7a presents the refined octree defined in Figure 3, where all the leaves of the computational tree are colored in red. To compute the distance from a point *P* to ω, $OL_{P}$ is determined and drawn in green in Figure 7b. All the bounding boxes of fibers immersed in this leaf are browsed to determine $d_{P}$ and $C_{P}$. The octree leaves intersecting this circle are determined and asterisked in Figure 7c. The intersection between the bounding boxes of fibers contained in those leaves and $C_{P}$ is examined, and if, and only if, an intersection is found, the distance from *x* to the fiber is determined. The same procedure is followed for points *Q* and *R*. Those three examples depict the efficiency of the method in different situations (the most frequent situation is the one described by the point *R*), where the number of evaluations of distances to elements is largely reduced. Table 2 shows a large decrease despite the low number of fibers immersed, which reduces the efficiency of the method. The octree construction and closest leaves determination costs are not included in this situation. However, the recursive construction and the distance to bounding boxes determination are cheap computationally compared to the distance to fibers evaluation, which requires projections. When a deeper octree is used for much bigger ω, evaluating distances to fibers become quite expensive, and savings brought by the octree rise rapidly.

### 3.2. Parallel Anisotropic Mesh Adaptation

Octree-optimized mesh immersion procedure is an efficient way to represent geometries if an accurate computational mesh is used as Section 3.1 stated. The results obtained with this technique are highly dependent on the position of the points, particularly at the interfaces. To that extent, a coupling between mesh immersion and the automatic generation of an anisotropic mesh is proposed in order to reduce the size of the problem to be treated. This iterative process starts with a coarse initial mesh, where geometries are immersed and reconstructed using the methods proposed in Section 3.1. A-posteriori error estimator [13,14] evaluates errors from the level-set results at each computational point, using the smoothed Heaviside function $H_{\varepsilon }$ described in Equation (2). In order to generate an anisotropic mesh, a tensor is defined at each point, enabling to measure the errors along each dimension. In other words, at each computational point, the variation of the function $H_{\varepsilon }$ along each direction is observed.

The adaptation relies on a uniform distribution of the error along the edges of the mesh in all the computational domain. A metric can be built, which allows to deform the mesh in order to attain uniform error: refinement is performed in the areas where the error is too important, while mesh is coarsened where low error is observed. As $H_{\varepsilon }$ is defined from a hyperbolic tangent, major gradients variation are found around the interfaces while the function is almost constant far from the frontiers. Consequently, around the interfaces, low edges are required to attain errors equivalent to the one obtained with large edges where gradients are almost null. Consequently, the new mesh will feature more nodes in the interest zones, and the reconstruction will gain precision. As the metric is built as a tensor, different stretching factors are used for each direction, which guarantees anisotropic meshing.

After several iterations, the errors are uniformly dispersed in the computational domain. Nodes are mostly concentrated around Γ, and the immersed geometry is well described. Highly-stretched mesh cells can be found in regions where very thin description is needed in one dimension while the others do not require particular attention. However, the stretching ratio of the mesh cells is limited, in order to ensure convergence of computations. The automatic and anisotropic mesh adaptation brings versatility, and at the same time guarantees that the results obtained with the mesh immersion procedure will be accurate. The reduction in the number of points required for the reconstruction enables to reduces both memory usage and computational costs. Coupled with an octree, an efficient optimization of the reconstruction is obtained. Moreover, this reconstruction process is executed on a multi-cores context in order to be able to combine the optimizations related to the use of mesh adaptation and octree with massively parallel computing. The parallelization of the process is performed by an iterative coupling between operations of independent adaptive mesh in different partitions and displacement of the interface between these partitions [15,16].

### 3.3. Weak Scalability Test of the Proposed Reconstruction Approach

To determine the scaling capability of the whole reconstruction procedure, weak scaling tests have been performed on the western French region, Pays de la Loire cluster Liger (a BULL/Atos DLC720 cluster, 6384 cores Intel Xeon (Haswell and Cascade Lake) (compute and visualization parallel procedures), a total of 36,608 Gigabytes of system memory, 5.33 GB per core, FDR Infiniband interconnect (56 GB/s)). Five microstructures were generated, as described previously, while keeping the same geometrical characteristics of fibers. To realize tests with similar workload per processor, the size of the computational domain and the number of immersed fibers were proportionally increased according to the number of the used cores, as detailed in Table 3.

**Figure 8:**
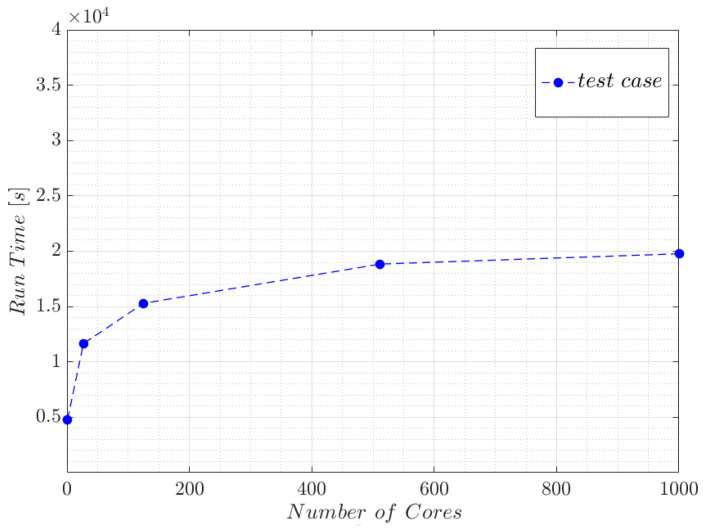
Total reconstruction time evolution as a function of used cores for the different test cases.

The reconstruction process started from an initial coarse mesh and took 30 iterations with constant precision and fixed octree parameters. For the different test cases, an average number of mesh nodes per core equal to $3\times 10^{5}$ was maintained with the exception of test 1 ($1.8\times 10^{5}$ nodes) where the volume of fibers that extend outside the computational domain and are therefore sliced is significant, so leading to a decrease in the number of nodes. Total time of the immersion and adaptation process as a function of the number of cores is represented in Figure 8. For an ideal weak scale test, the run time is expected to stay constant while the workload is increased in direct proportion to the number of processors. For real case, as shown in Figure 8, a deviation can be observed due to communications and partitioning efforts. However, according to the same figure, the running time variation is relatively small between the tests (except for the first one where the workload is different) which allows to consider that for a scaled problem size, the domain reconstruction approach has good efficiency in terms of weak scalability.

## 4. Flow Simulation Examples: Application to Permeability Computation

### 4.1. Flow Simulation

The resulting mesh from the reconstruction process can be used to simulate various physical phenomena, such as those involved in fluid-structure interaction problems. Generally, for composite flow applications, incompressible Stokes flow around the fibers is considered. By considering a stationary regime and neglecting the volume forces, the variational form of the Stokes problem for velocity field, **u**, and pressure field, *p*, is written:

(4) $$\begin{array}{c} \left(v,q\right)\;\in V_{0}\times Q\\ \left\{\begin{array}{c} {\left(2\eta \varepsilon \left(u\right):\varepsilon \left(v\right)\right)}_{\Omega }-{\left(p,\nabla \cdot v\right)}_{\Omega }=0\\ {\left(\nabla \cdot u,q\right)}_{\Omega }=0 \end{array}\right. \end{array}$$

where ε is the strain rate tensor.

A monolithic approach is used, i.e., the flow Equation (4) are solved on the single mesh defined over the whole computational domain, Ω, regardless of the type of phase it contains. The different phases are distinguished by their physical properties which are taken into account through a mixing law. A linear mixture relation is used for the viscosity, η, and described by the Equation (5).

(5) $$\eta =\eta_{f}H_{\epsilon }+\eta_{s}\left(1-H_{\epsilon }\right)$$

$\eta_{f}$ and $\eta_{s}$ are, respectively, the viscosities of the liquid and solid phases. $\eta_{s}$ acts as a penalty parameter: when it is high enough, shear rate in the penalized phase becomes close to zero and we find a rigid body motion. This is a simple way to obtain results similar to those provided by an augmented Lagrangian method where a Lagrange multiplier is used to impose a constraint on the solid phase to avoid its deformation [17]. To solve the system (4) using a finite element method, a stabilized approach of VMS type is employed [12]. The used software in this work is ICI-tech, developed at the High Performance Computing Institute (ICI) of Centrale Nantes and implemented for massively parallel context.

### 4.2. Permeability Computation Procedure

Predicting permeability is a very important issue in the field of composite forming process. However, it is tricky and complex to obtain experimentally and numerically reliable results, because most simulations are carried out in small periodic representative elementary volumes, under a lot of simplifying assumptions that idealize the real media. Here, we chose to rise to the challenge to numerically determine the permeability tensor of a large virtual sample of fibrous media that imitates sophisticated real media. In three-dimensional cases, permeability is characterized by a symmetric second-order tensor **K**. This tensor relates the average fluid velocity 〈u〉 to the average pressure gradient on the fluid domain ${\langle \nabla p\rangle }^{f}$, as shown by the Darcy law below:

(6) $$\langle u\rangle =-\frac{K}{\eta }{\langle \nabla p\rangle }^{f}$$

Using a monolithic approach with finite element discretization, the homogenized velocity and pressure fields are written as the sum of their integration on each mesh element $\Omega_{e}$ of the simulation domain Ω:

(7) $$\langle u\rangle =\frac{1}{V_{\Omega }}\sum_{e}\int_{\Omega_{e}}\left(1-H_{\epsilon }\left(\alpha \right)\right)u\;d\Omega_{e}$$

(8) $${\langle \nabla p\rangle }^{f}=\frac{1}{V_{\Omega_{f}}}\sum_{e}\int_{\Omega_{e}}\left(1-H_{\epsilon }\left(\alpha \right)\right)\nabla p\;d\Omega_{e}$$

where $V_{\Omega }$ is the volume of the total domain and $V_{\Omega_{f}}$ is the volume of the fluid domain.

To predict permeability, the proposed simulation procedure relies on microstructure generation, phase reconstruction, mesh adaptation, and resolution of the Stokes equations, considering that fibers are static and impermeable. In fact, to determine all components of **K**, three flows in the three directions *x*, *y*, and *z* are successively simulated, an exponent {1,2,3} is referred to each one. The flow is induced by an imposed pressure gradient. Depending on the direction where the flow is desired, a constant pressure field on the input face of the simulation domain against a null field on the output face is imposed. For the other faces of the domain, only the normal component of the velocity field is imposed as null. Assuming that the permeability tensor is symmetric and positive definite, its components can be calculated by the resolution of the overdetermined linear system given by:

(9) $$\left[\begin{array}{ccccccccc} {\langle \nabla p_{x}\rangle }^{1} & {\langle \nabla p_{y}\rangle }^{1} & {\langle \nabla p_{z}\rangle }^{1} & 0 & 0 & 0 & 0 & 0 & 0\\ 0 & 0 & 0 & {\langle \nabla p_{x}\rangle }^{1} & {\langle \nabla p_{y}\rangle }^{1} & {\langle \nabla p_{z}\rangle }^{1} & 0 & 0 & 0\\ 0 & 0 & 0 & 0 & 0 & 0 & {\langle \nabla p_{x}\rangle }^{1} & {\langle \nabla p_{y}\rangle }^{1} & {\langle \nabla p_{z}\rangle }^{1}\\ {\langle \nabla p_{x}\rangle }^{2} & {\langle \nabla p_{y}\rangle }^{2} & {\langle \nabla p_{z}\rangle }^{2} & 0 & 0 & 0 & 0 & 0 & 0\\ 0 & 0 & 0 & {\langle \nabla p_{x}\rangle }^{2} & {\langle \nabla p_{y}\rangle }^{2} & {\langle \nabla p_{z}\rangle }^{2} & 0 & 0 & 0\\ 0 & 0 & 0 & 0 & 0 & 0 & {\langle \nabla p_{x}\rangle }^{2} & {\langle \nabla p_{y}\rangle }^{2} & {\langle \nabla p_{z}\rangle }^{2}\\ {\langle \nabla p_{x}\rangle }^{3} & {\langle \nabla p_{y}\rangle }^{3} & {\langle \nabla p_{z}\rangle }^{3} & 0 & 0 & 0 & 0 & 0 & 0\\ 0 & 0 & 0 & {\langle \nabla p_{x}\rangle }^{2} & {\langle \nabla p_{y}\rangle }^{2} & {\langle \nabla p_{z}\rangle }^{3} & 0 & 0 & 0\\ 0 & 0 & 0 & 0 & 0 & 0 & {\langle \nabla p_{x}\rangle }^{3} & {\langle \nabla p_{y}\rangle }^{3} & {\langle \nabla p_{z}\rangle }^{3}\\ 0 & 1 & 0 & -1 & 0 & 0 & 0 & 0 & 0\\ 0 & 0 & 1 & 0 & 0 & 0 & -1 & 0 & 0\\ 0 & 0 & 0 & 0 & 0 & 1 & 0 & -1 & 0 \end{array}\right]\left[\begin{array}{c} K_{xx}\\ K_{xy}\\ K_{xz}\\ K_{yx}\\ K_{yy}\\ K_{yz}\\ K_{zx}\\ K_{zy}\\ K_{zz} \end{array}\right]=-\eta \left[\begin{array}{c} {\langle u_{x}\rangle }^{1}\\ {\langle u_{y}\rangle }^{1}\\ {\langle u_{z}\rangle }^{1}\\ {\langle u_{x}\rangle }^{2}\\ {\langle u_{y}\rangle }^{2}\\ {\langle u_{z}\rangle }^{2}\\ {\langle u_{x}\rangle }^{3}\\ {\langle u_{y}\rangle }^{3}\\ {\langle u_{z}\rangle }^{3}\\ 0\\ 0\\ 0 \end{array}\right]$$

The solution obtained from the resolution of this matrix system (9) is, obviously, an approximate solution. To ensure a perfect symmetry of **K**, if necessary, the following modification to the extra diagonal terms is made:

(10) $$K_{ij}^{final}=K_{ji}^{final}=\frac{K_{ij}+K_{ji}}{2}$$

### 4.3. Permeability Computation Validation

**Figure 9:**
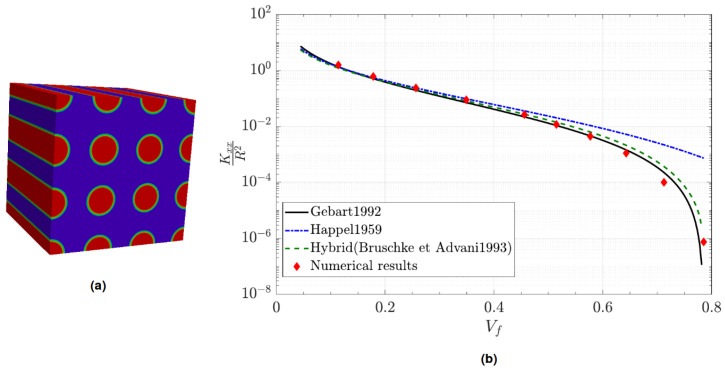
Comparison of computed permeability with some analytical models: (**a**) Simulated parallel square packing configuration. (**b**) Normalized transverse permeability results.

To validate permeability computation, the whole procedure was applied to a parallel square packing of fibers having an identical diameter. Rigidity of fibers was ensured by imposing $\eta_{s}=500\eta_{f}$ and a zero velocity condition was imposed upon them. Figure 9a shows the used geometry configuration for $V_{f}=25.65\%$. Equation (11) represents its calculated permeability tensor adimensionalized by the square of fiber radius which respect a transverse isotropic form as expected from the symmetry of the packing.

(11) $$K=\left[\begin{array}{ccc} 0.21 & 0 & 0\\ 0 & 0.21 & 0\\ 0 & 0 & 0.28 \end{array}\right]$$

Permeability evolution according to fiber volume fraction was studied by varying fiber diameter and keeping same the domain size for all simulations. The obtained results of normalized transverse permeability are reported in Figure 9b and compared to the model of [18,19,20]. The observed permeability values through this graph are in the same order than the one obtained from analytical laws which is relevant to our approach.

### 4.4. Application for 10,000 Fibers

#### 4.4.1. Microstructure Generation

The first step of the process is the microstructure generation using the octree optimized algorithm described in Section 2. A sample of approximately 10,000 (exactly 10,062) collision-free fibers is created in a cubic domain with an edge length of 1.35 mm. The fibers have a common diameter of 15 μm and a length that follows a normal distribution of mean 0.2 mm and standard deviation 0.03 mm. The obtained volume fraction is $V_{f}=14\%$. The orientation state is nearly isotropic and is given by the following orientation tensor $a_{2}$ [21]:

$$a_{2}=\left[\begin{array}{ccc} 0.334241 & -0.00219696 & -0.018116\\ -0.00219696 & 0.34166 & -0.00620966\\ -0.018116 & -0.00620966 & 0.324099 \end{array}\right]$$

**Figure 10:**
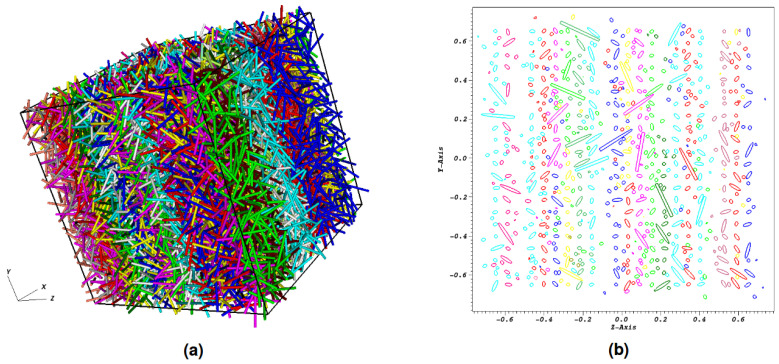
Studied generated microstructure: (**a**) 10,000 generated fibers. (**b**) A random slice showing no collisions.

Figure 10 shows the set of the generated fibers. Despite the fact that the generation is sequential, these fibers are created in only **1min44s** thanks to the octree contribution.

#### 4.4.2. Microstructure Reconstruction with Adaptative Mesh

**Figure 11:**
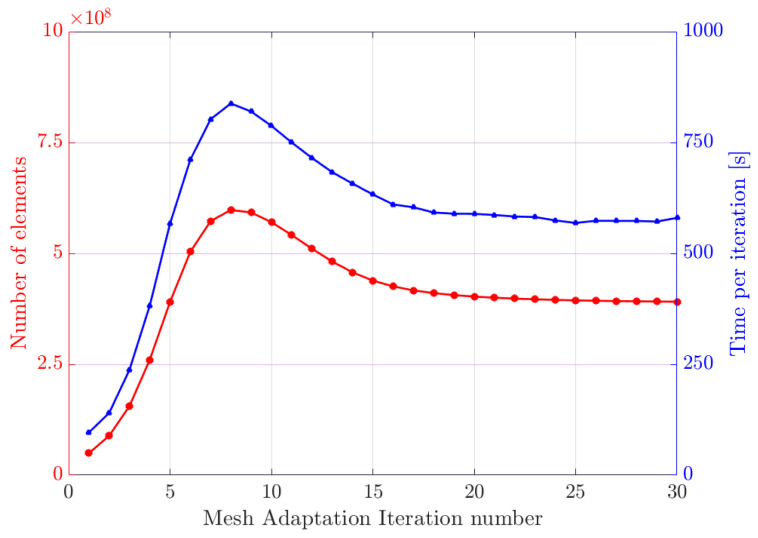
Evolution of the mesh number of elements (**left** axis) and calculation cost (**right** axis) during the 30 iterations of adaptation of anisotropic mesh, performed on 384 cores.

The computation was performed on 384 cores. Starting from an initial mesh of ≈4.6 million nodes and ≈27 million elements, after 30 iterations, an adapted final mesh of ≈67 million nodes and ≈391 million elements is created by the methods described in Section 3.1 and Section 3.2. For $H_{\varepsilon }$ with ε = 3.125 μm, the total immersion and adaptation process required **4h52min** for the 30 iterations. Figure 11 shows the evolution, in a number of elements for each iteration of the mesh adaptation, as well as the computational time. During the first iterations of immersion of the generated fibers in the initial mesh, the mesher adds a considerable number of elements until reaching a peak at the ninth iteration, in order to properly capture the geometries of all the fibers at first. Then, the mesher focuses its work on optimizing the mesh adaptation at the interfaces while respecting a criterion of mesh quality. Once an efficient mesh is achieved, the number of elements stabilizes. The time evolution curve naturally follows the evolution of the mesh size.

#### 4.4.3. Flow Resolution and Permeability Tensor Computation

**Figure 12:**
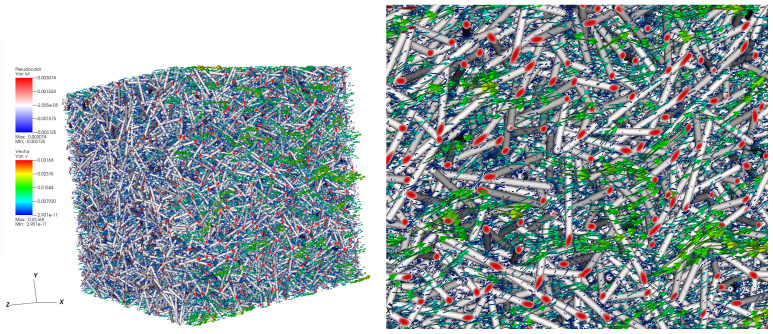
Flow according to *x* direction: (**a**) velocity vector around the fibers. (**b**) Zoom around a zone of the figure.

Three pressure gradients are applied to the constructed finite element mesh in order to generate the flows required for the identification of **K**. Figure 12 shows the pressure field and velocity vectors around the immersed fibers for the flow in the *x* direction. These results were obtained for a resolution time of the system (4) equal to approximately **7min** minutes on 384 CPUs.

The predicted full permeability tensor adimensionalized by the square of fiber radius for this media is as follows:

$$K=\left[\begin{array}{ccc} 0.7322 & -0.0013 & -0.0033\\ -0.0013 & 0.7444 & -0.0025\\ -0.0033 & -0.0025 & 0.7089 \end{array}\right]$$

For isotropic material, only the three diagonal elements are non-null and they are equal. Here, the studied sample is nearly isotropic. For this reason, the obtained diagonal elements are quite similar and the off-diagonal elements are smaller by around two orders of magnitude.

## 5. Conclusions

Obtained results show our capability thanks to an octree implementation to deal with big data in terms of input of permeability simulation and to perform reliable finite element calculation on complex geometries. Through the proposed method, further studies can be conducted to better quantify the impact of the microstructural parameters on the permeability and, thus, avoiding problems related to the choice of the size of the simulation domains, which remains rather delicate to define, especially in the case of non-periodic geometries. We can also think about exploring the permeability of multiaxial tissues of the non-crimp fabric (NCF) or textile type. Thanks to the several numerical optimization, the permeability can thus be evaluated at the microscopic scale on several layers by representing the fibers inside the wicks.

## Figures and Tables

**Table 1:** Nomenclature used to discuss distance computation algorithm.

Variable Name	Signification
*x*	Point of computational mesh
ω	Shape immersed represented by elements
$E_{x}$	Closest element of the set representing ω from *x*
$OL_{x}$	Closest octree leaf from *x*
$d_{x}$	Maximal theoretical distance from *x* to $E_{x}$
$C_{x}$	Circle/sphere of center *x* and radius $d_{x}$
$\alpha_{x}$	Signed distance from *x* to $E_{x}$

**Table 2:** Reduction in the number of evaluations of distances provided by the octree.

Distances to Fiber/Points	*P*	*Q*	*R*	Total
no octree	14	14	14	42
octree	3	3	1	7

**Table 3:** Simulation parameters for weak scalability test performed on liger supercomputer.

	Number of Fibers	Domain Edge Size	Number of Cores	Total Mesh Nodes
test 1	8	0.178	1	172,245
test 2	216	0.534	27	728,895
test 3	1000	0.890	125	37,153,365
test 4	4096	1.425	512	160,374,769
test 5	8000	1.781	1000	317,813,266
